# Stereotype-driven emotional responses and their impact on discriminatory intentions towards suicidal individuals

**DOI:** 10.1186/s40359-024-01633-9

**Published:** 2024-03-15

**Authors:** Hannah Lee, Soontae An

**Affiliations:** 1 Ewha Institute for Age Integration Research, Ewha Womans University, #504-1, SK Telecom Building, 52 Ewha Yudae Gil, Seodaemun-Gu, Seoul, 03760 Korea; 2 Department of Communication and Media, Ewha Womans University, Seoul, South Korea

**Keywords:** Suicide stigma, Stigma reduction, Suicide prevention, Emotional responses

## Abstract

**Background:**

This research delves into the role of stereotypes and emotional prejudice in behavioral intentions, particularly towards individuals with suicidal tendencies. The study extends the cognitive-affective-behavioral process model, identifying pathways that negative stereotypes use to impact emotional responses and behavioral intentions.

**Methods:**

A cross-sectional online survey was conducted in South Korea, utilizing the largest Korean online panel (1,623,938 users) to recruit 552 eligible participants (49.1% male, 50.9% female) aged 20 and above with online access and no history of suicide attempts. The survey assessed negative stereotypes, prejudices, and behavioral intentions related to suicidal thoughts, employing specific measurements.

**Results:**

The findings established the correlation between negative stereotypes and both stigmatized emotional responses and discriminatory intentions. The study uniquely demonstrated that emotional responses act as a bridge between negative stereotypes and behavioral intentions towards suicidal individuals. These findings carry profound implications for health psychology, emphasizing the necessity of modifying attitudes to reduce suicide stigma. It was observed that stereotypical perceptions fuel negative emotions, which in turn provoke various behavioral intentions.

**Conclusions:**

The study enhances our understanding of the influential role emotional reactions can have in shaping attitudes. It points towards the potential that addressing emotions holds in the stigma process, enabling people to shift their attitudes about stigmatized individuals, thus establishing intervention opportunities for stigma reduction in health psychology.

## Introduction

Globally, more than 2,000 lives are lost to suicide every day [1]. With each suicide death, there are numerous additional suicide attempts. In Korea alone, nearly 80 individuals make an attempt on their own life each day but survive [2]. Survivors of suicide attempts face twice the risk of future suicidal behavior compared to those who have not attempted suicide. Moreover, the rising number of Koreans suffering from depression indicates that countless individuals struggle with severe suicidal ideation [2]. For these individuals, stigma is a significant concern. Although some people experiencing suicidal ideation feel the need to seek help, most are afraid and hesitant to talk about their symptoms [3], as disclosing suicidality may lead to stigmatization and social rejection [4].

The stigma attached to suicide attempts is a crucial barrier to suicide prevention. Individuals who have survived suicide attempts often feel guilty and shame [5] and avoid discussing their experiences [6]. Across various cultural contexts, most societies typically regard those with suicidal ideation as social deviants. For instance, in Korea, suicidal behavior is seen as a sign of weakness and immorality rather than a public health issue [7–9]. In such environments, it is unsurprising that those with suicidal ideation cannot rely on informal support networks for emotional support or appropriate advice. When individuals conceal their suicidal thoughts and suppress psychological distress, they are unable to access the benefits of formal and informal support networks [10, 11]. Therefore, reducing suicide stigma should be the primary strategy for successful suicide prevention.

This study, part of a larger project exploring stigma reduction strategies, aims to examine how to decrease discrimination toward stigmatized individuals and promote help-giving. Prior research has shown strong evidence that people considering suicide face discrimination in the form of distancing, ignoring, mistrust, and shaming [3, 12, 13]. However, it remains unclear how the public develops stigmatizing attitudes toward those with suicidal ideation and why these attitudes lead to discriminatory behaviors against persons with suicidal ideation. In addition, no study has yet examined the associations between perceived suicide stigma and stigma-related behavioral outcomes from the general public’s perspective. Understanding how suicide stigma relates to affective and behavioral reactions is essential to gain insights into stigma-reduction strategies.

The purpose of this study is to conduct a theory-based investigation into specific cognitive attributions, emotional reactions, and behavioral intentions toward suicidal individuals. Suicide stigma refers to negative attitudes, beliefs, and behaviors directed toward those in society who struggle with mental health problems such as suicidal thoughts [14]. Stigma is a complex construct encompassing negative attitudes and behaviors targeting particular groups [15]. Corrigan et al. [16] described the concept of stigma through a social-cognitive model consisting of three components: stereotypes, prejudices, and discriminations. Stereotypes embody the cognitions held and shared by the public regarding stigmatized objects. When individuals conform to these stereotypes, prejudices may form, which are emotionally driven assessments that elicit automatic responses. Prejudice can lead to discriminatory behaviors, such as avoidance or disdain, which significantly reduce the chances for stigmatized individuals to seek help.

Drawing on the concept of stigma, this study examines the pathway of negative stereotypes on emotional and behavioral reactions toward individuals with suicidal thoughts. Corrigan et al. [16] suggested a comprehensive cognitive-emotional process model that provides a theoretical explanation for emotions and behavioral reactions related to suicide. Rudolph et al. [17] demonstrated the causal cognition-emotion-behavior sequence through a meta-analytic test, showing that judgments of responsibility determine emotional reactions of anger and sympathy, which in turn directly influence help-giving and aggression. However, few empirical studies have specifically examined how aspects of the cognitive-emotional process model relate to helping or discriminatory behaviors toward people with suicidal issues.

While previous studies have emphasized the role of stereotypes in shaping behavioral reactions, this study focuses on the role of emotional responses toward individuals with suicidal thoughts. According to the cognitive-emotional process model [16], emotions serve as antecedent factors for behavioral intentions, and negative emotions such as anger can trigger aggressive or avoidant behavior. In this context, people’s feelings toward suicide or those who have attempted suicide also ultimately influence their behavior toward these individuals [18]. By incorporating various emotional reactions into this model, this research aims to understand the relationships between the negative stereotypes, emotional responses, and behavioral intentions toward individuals experiencing suicidal ideations. Specifically, this study seeks to investigate the different mechanisms of behavioral intention changes based on varying emotional reactions.

The present study is anchored on Corrigan et al.’s stigma path model [16] and aims to elucidate the intricate relationship between stereotypes, emotional reactions, and behavioral intentions towards individuals with suicidal ideation. Accordingly, we propose the following hypotheses:

- Hypothesis 1: Negative stereotypes about individuals with suicidal ideation, such as perceptions of incompetence, selfishness, and immorality, will be significantly associated with negative emotional reactions, which include anger, fear, shame, and disgust.
- Hypothesis 2: These emotional reactions will, in turn, mediate the relationship between negative stereotypes and behavioral intentions. Specifically, negative emotions are hypothesized to lead to adverse behavioral intentions such as avoidance, disdain, coercion, and a reduced willingness to provide help to individuals with suicidal tendencies.

By testing these hypotheses, this study intends to deepen the understanding of the cognitive-affective-behavioral process underlying stigma towards suicidal individuals. The research model depicting these relationships is illustrated in the figure below. This model, based on Corrigan et al.’s stigma path model [16], has been instrumental in understanding the stigma process across various contexts and is particularly relevant to our study's focus on suicidal ideation.

## Methods

### Participants and recruitment procedures

A cross-sectional online survey was conducted in South Korea by a professional research firm managing the largest Korean online panel, which includes 1,623,938 users. This firm was tasked with recruiting and screening eligible participants—individuals aged 20 and over with online access and no history of suicide attempts. Invited participants received URLs via email, which provided them access to the survey. The panel company’s server performed a randomized email blast to all panel members to inquire about their willingness to participate in this study. Interested individuals were directed to an initial webpage via the provided link. This webpage conveyed essential information about the study’s objectives, assured participants of the confidentiality and security of their data, warned about potentially distressing questions, displayed the ethical clearance, and offered emergency contact information. To ensure that all participants had no history of suicide attempts, a self-report question was included at the beginning of the survey, explicitly asking participants if they had ever attempted suicide.

To determine an adequate sample size for the study, a power analysis was conducted. The analysis was informed by the expected effect sizes derived from preliminary studies and the complex model involving multiple variables. Based on these calculations, utilizing G*Power software, it was determined that a sample size of 500 would be optimal. This size would ensure sufficient power (0.80) to detect a medium effect size at an alpha level of 0.05 in our multiple regression analysis, accounting for the number of predictors and the potential for dropouts and non-response. This measure was taken to ensure the robustness and reliability of our findings.

For validating the online survey, several measures were implemented. The survey platform was programmed to track and maintain the consistency of responses, flagging any irregularities or patterns that suggested invalid responses. Additionally, attention-check questions were strategically placed throughout the survey to gauge participants' attentiveness and the sincerity of their responses. Furthermore, prior to widespread dissemination, the researchers conducted thorough testing of the survey link. This testing phase involved the research team members themselves filling out the survey to verify that the link was functioning correctly under various scenarios. All identified issues, such as broken links, loading errors, or unclear instructions, were documented and corrected. This proactive approach was taken to preempt any technical difficulties that could arise for participants, thus ensuring the integrity and seamless administration of the survey process.

### Ethical considerations

Ethical considerations were of utmost importance in our study. We secured ethical approval from our institutional review board before conducting the survey, which included the strategy for confirming that participants had no history of suicide attempts. Given the sensitive nature of this screening question, we exercised considerable care to pose it in a non-intrusive way, and we ensured that resources for mental health support were readily available at both the beginning and end of the survey. All participants were offered financial incentives for their time and contribution to the study.

### Demographics

A total of 552 respondents participated (male: N = 271, 49.1%; female: N = 281, 50.9%), with an average age of 44.38 years (SD = 13.71). The majority of participants (76.1%) had completed college or obtained a bachelor’s degree. Most participants (82.3%) exhibited normal depression levels, as assessed by the PHQ-9 [19], and nearly four-fifths of participants (77.9%) reported having an acquaintance who had attempted or died by suicide (see Table 1).

**Table 1:** Participants’ characteristics (*N* = 552)

Variables		*N*	%
Gender	Male	271	49.1
	Female	281	50.9
Age	20–29 years	108	24.9
	30–39 years	105	24.3
	40–49 years	109	25.7
	50–59	113	25.1
	60-over years	117	21.2
Education level	Less than high school	6	1.1
	High school graduate	84	15.2
	College/University student	42	7.6
	College/University graduate	354	64.1
	Master/Doctor degree	66	12.0
Yearly House Income	under $25,000	175	31.7
	$25,000-$34,999	124	22.5
	$35,000-$49,999	98	17.8
	$50,000-$74,999	102	18.5
	$75,000-$9,999	38	6.9
	$100,000-$149,999	13	2.4
	$150,000 above	2	0.4
Depression Level	Normal	309	56.0
	Minimal depressive symptoms	145	26.3
	Mild Severity	66	12.0
	Moderate Severity	17	3.1
	Severe Severity	15	2.7
Acquaintance Suicide (attempted from suicide)	Yes	122	22.1
	No	430	77.9

### Measures

This online survey assessed general sociodemographic information and specifically focused on three key topics: (a) negative stereotypes pertaining to awareness and diagnoses related to individuals experiencing suicidal thoughts, (b) prejudices surrounding stigmatized emotional responses toward those contemplating suicide, and (c) behavioral intentions concerning individuals struggling with suicidal ideation. The measurements used in the survey are described below.

#### Negative stereotypes

To evaluate participants' perceptions and attitudes towards suicidal individuals, we employed the Korean Suicide Stigma Scale (KSSS) [20]. The KSSS is a newly developed measure designed to capture two components of suicide stigma: stereotype and prejudice. Additionally, this scale is for Asian society, modifying a culturally focused design based on Corrigan et al.’s [14] and Batterham et al.’s [21] stigma scale. We adapted 12 items from the KSSS to measure negative stereotypes consisting of three subfactors. The first subfactor, the incompetence factor contained four items (Cronbach’s α = 0.865) with adjectives such as “lacking self-control,” “weak,” “reckless,” and “irresponsible.” The second, selfishness measured four items (Cronbach’s α = 0.859), such as “cruel,” “selfish,” “self-serving,” and “unfilial.” The last subfactor, immorality, was measured with four items (Cronbach’s α = 0.880), including “sinful,” “immoral,” “reprehensible,” and “barbaric.” Each item measured the degree of stereotypes toward suicidal individuals using a 5-point Likert scale ranging from 1 (strongly disagree) to 5 (strongly agree). In analyzing the data, we calculated the average score for the items measuring each subfactor. These average scores were then utilized in subsequent analyses, with higher averages indicating higher levels of negative stereotypes toward suicidal individuals.

#### Emotional prejudice

We adapted two factors (i.e., disgust and shame) from the KSSS to measure emotional prejudice. Participants responded to four items assessing *disgust* toward suicidal individuals (Cronbach’s α = 0.943; adapted from An & Lee [20]; “dark,” “nauseated,” “disgrace,” “burdensome”). The *shame* factor contained four items (Cronbach’s α = 0.920; adapted from An & Lee, [20]; “shallow,” “shameful,” “disapproving,” “troublesome”). To explore the effects of various types of emotions, fear and anger were added as factors regarding emotional prejudice. We adopted a subscale of the Suicide Stigma Assessment Scale (SSAS; Corrigan et al., [14]) to measure *fear* (Cronbach’s α = 0.837; “afraid,” “fears,” “doubts truthfulness”) and *anger* (Cronbach’s α = 0.742; “angry,” “frustrated,” “embarrassed”). All items utilized a 5-point Likert response scale ranging from 1 (strongly disagree) to 5 (strongly agree). For analysis, we computed the mean value of the items representing each subfactor.

#### Behavioral intentions

We dealt with four variables to measure behavioral intention toward suicidal individuals. According to the SSAS of Corrigan et al. [14], discriminatory intents toward stigmatized persons could be categorized into three factors: avoidance, disdain, and coercion. To measure three behavioral dimensions, we adopted and modified the SSAS [14]. The original SSAS used to measure discrimination originally consisted of 13 items for the avoidance factor, 4 items for the disdain factor, and 5 items for the coercion factor. We utilized the SSAS [14] to measure *avoidance*, which consists of 5 items (Cronbach’s α = 0.948; sample items “people stay away,” “people won’t have serious relationships with,” “people keep distance”); *disdain*, comprising 2 items (Cronbach’s α = 0.785; sample items “people are cautious near them,” “people gossip”); and *coercion*, including 4 items (Cronbach’s α = 0.810; sample items “hospitalize them,” “force them to take meds,” “lock them up,” “keep them under a microscope”). Participants responded on 5-point scales (1 = strongly disagree, 5 = strongly agree), with higher scores indicating greater discriminatory intention. Additionally, participants answered two items assessing their *help-giving* intention toward suicidal individuals, adapted and modified from Weiner et al. [22]. The two items (Cronbach’s α = 812) were “I am willing to assist a suicidal person” and “If an individual wishes to commit suicide, it is their personal matter, and we don’t need to help them (reverse coded).” Responses were given on a 5-point scale (1 = strongly disagree; 5 = strongly agree). The analysis was conducted by calculating the mean values of the items that measure each subfactor.

#### Depression level

The severity of depression symptoms was measured using the Patient Health Questionnaire-9 [18]. This scale (as outlined in the DSM-IV, APA, 2000) contains nine items (Cronbach’s α = 0.906, e.g., “Little interest or pleasure in doing things,” “Trouble falling or staying asleep, or sleeping too much”), assessing the frequency of the depressive symptoms they had experienced in the two weeks prior to the survey. All items were measured on a 4-point scale ranging from 0 (not at all) to 3 (nearly every day). The sum scores “below 5,” “5–9,” “10–14,” “15–19,” and “ ≥ 20” represent “normal,” “minimal,” “mild,” “moderate,” and “severe” depression, respectively.

#### Contact experience with suicide

Participants were queried about their personal experience with a question that asked, “Has anyone close to you (e.g., a family member or friend) ever attempted suicide?” The response options available were “Yes” or “No.”

### Data analysis

Prior to testing our main hypotheses, preliminary analyses were conducted to examine the descriptive statistics and correlations among all study variables. Following this, the main analysis to test our multiple mediation model was carried out using the Hayes PROCESS macro (Model 4). All variables were standardized before the model was tested. The parameters were estimated using the bootstrap method with 5,000 samples and a 95% confidence interval (CI) using the bias-corrected percentile method. The parameters are significant if the CI does not include zero. All analyses were conducted using SPSS version 26.0.

## Results

### Bivariate correlations

Table 2 displays the correlations among the key variables. In terms of demographics and stigma components, gender exhibited significant correlations with incompetence (*r* = -0.18, *p* < 0.001), selfishness (*r* = -0.09, *p* = 0.032), immorality (*r* = -0.22, *p* < 0.001), shame (*r* = -0.16, *p* < 0.001), disgust (*r* = -0.19, *p* < 0.001), avoidance (*r* = -0.14, *p* = 0.001), disdain (*r* = -0.12, *p* = 0.004), and help-giving (*r* = 0.11, *p* = 0.011). Age showed positive correlations with incompetence (*r* = 0.22, *p* < 0.001), selfishness (*r* = 0.28, *p* = 0.032), immorality (*r* = 0.25, *p* < 0.001), fear (*r* = 0.12, *p* = 0.005), anger (*r* = 0.20, *p* < 0.001), shame (*r* = 0.28, *p* < 0.001), disgust (*r* = 0.20, *p* < 0.001), and help-giving (*r* = 0.15, *p* = 0.001). Religious belief was negatively correlated with shame (*r* = -0.10, *p* = 0.026) and help-giving (*r* = -0.10, *p* = 0.014). Depression level was negatively correlated with incompetence (*r* = -0.19, *p* < 0.001), shame (*r* = -0.14, *p* = 0.001) and help-giving (*r* = -0.09, *p* = 0.027). Contact experience with suicide was positively correlated with incompetence (*r* = -0.09, *p* = 0.027) and avoidance (*r* = 0.08, *p* = 0.047). Considering the results of the correlations, five characteristics of the participants were chosen as control variables (i.e., gender, age, religious beliefs, depression symptoms, and contact with suicide).

**Table 2:** Correlations between key variables

	1	2	3	4	5	6	7	8	9	10	11	12	13	14	15	16	17	18
1. Gender	1																	
2. Age	.01	1																
3. House Income	-.17**	.20**	1															
4. Education	-.04	.01	.32**	1														
5. Religious Beliefs	-.11**	-.21**	-.01	-.06	1													
6. Depression Level	.01	-.13**	-.11*	-.01	.08	1												
7. Contact Experience	.02	-.07	.00	.00	-.01	-.12**	1											
8.Incompetence	-.18**	.22**	.03	-.04	-.05	-.19**	.09*	1										
9. Selfishness	-.09*	.28**	.08	.01	-.04	-.07	.03	.60**	1									
10. Immorality	-.22**	.25**	.06	.01	-.08	-.07	.07	.62**	.69**	1								
11. Fear	-.03	.12**	.08	.05	-.07	.07	.06	.20**	.18**	.26**	1							
12. Anger	-.05	.20**	.04	-.01	-.07	-.02	.01	.36**	.43**	.43**	.39**	1						
13. Shame	-.19**	.28**	.04	-.03	-.10*	-.14**	.05	.74**	.58**	.72**	.23**	.44**	1					
14. Disgust	-.16**	.20**	.05	.03	-.06	-.06	.01	.55**	.52**	.78**	.21**	.38**	.79**	1				
15. Avoidance	-.14**	-.01	.00	.03	.03	.05	.08*	.26**	.24**	.35**	.43**	.39**	.36**	.40**	1			
16. Disdain	-.12**	-.06	-.04	.03	.04	.06	.05	.17**	.14**	.30**	.34**	.36**	.30**	.39**	.76**	1		
17. Coercion	-.02	.04	-.02	-.02	-.03	.06	.06	.20**	.21**	.27**	.35**	.39**	.24**	.28**	.47**	.54**	1	
18. Help-giving	.11*	.15**	-.07	-.03	-.10*	-.09*	.05	.00	.03	-.08*	-.12**	-.07	-.12**	-.20**	-.23**	-.29**	-.06	1

*N* = 552

^*^p < .05

^**^p < .01

### Multiple mediation analysis

**Fig. 1:**
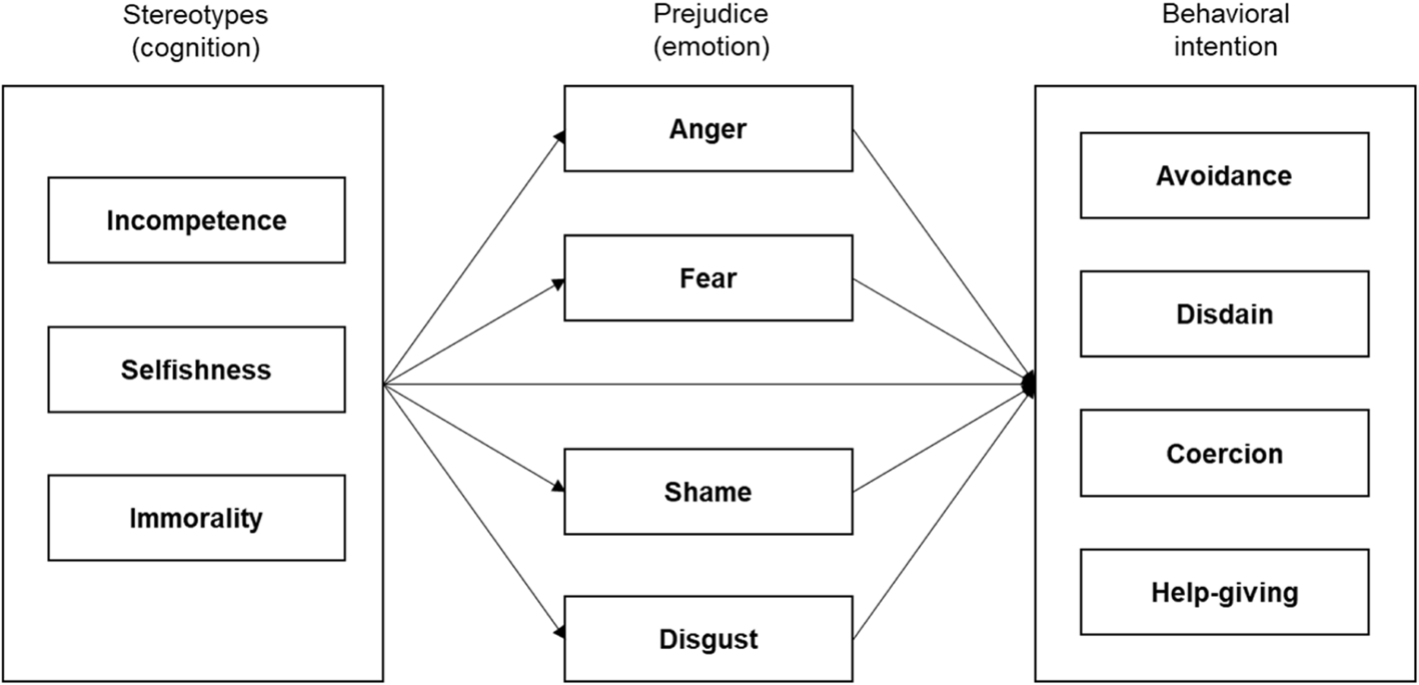
Research model based on Corrigan et al.’s Stigma Path Model. *Note.* Figure 1 illustrates the hypothesized relationships between stereotypes, emotional reactions, and behavioral intentions towards individuals with suicidal ideation. This model is adapted from Corrigan et al. [16]

This study used the PROCESS macro (model 4) to test whether four types of emotional reactions (i.e., anger, fear, disgust, and shame) mediated the relationship between negative stereotypes and behavioral intentions toward a suicidal person. Multiple mediation analysis offers the advantage of being able to test the indirect effect through four emotional reactions while concurrently controlling for the influence of the other variables. As depicted in the research model (see Fig. 1), we carried out PROCESS analysis with three subfactors of negative stereotypes (i.e., incompetence, selfishness, and immorality) and four dependent variables as behavioral intentions (i.e., avoidance, disdain, coercion, and help-giving). To estimate the direct and indirect effects of three independent variables on each of the dependent variables, one independent variable in the research model was designated as the X variable, while the remaining two variables were treated as covariates (p.196–197) [23]. To validate the mediation model index, we performed statistical analysis three times by alternating the positions of variables from the X variable to covariates. Gender, age, religious beliefs, depression symptoms, and contact experience with suicide were incorporated into the model as additional covariates.

### Path 1. The impact of three stereotypical perceptions on four stereotyped emotions (X → M)

Three stereotypical perceptions of suicidal individuals directly influenced emotional responses. Specifically, the incompetence factor was positively related to shame (*ß* = 0.47, t = 13.83, *p* < 0.001) and disgust (*ß* = 0.15, t = 3.98, *p* < 0.001). Selfishness exhibited a statistically significant positive influence on anger (*ß* = 0.22, t = 3.94, *p* < 0.001) and disgust (*ß* = 0.08, t = 2.16, *p* = 0.031). Immorality had significant impacts on fear (*ß* = 0.23, t = 3.69, *p* < 0.001), anger (*ß* = 0.21, t = 3.73, *p* < 0.001), shame (*ß* = 0.42, t = 11.30, *p* < 0.001), and disgust (*ß* = 0.75, t = 18.98, *p* < 0.001). The outcomes of these paths showed consistent results across all four mediation models in this study.

### Path 2. Direct effects of three stereotypical perceptions on each behavioral intention (X → Y)

We conducted a series of separate multiple mediation analyses, one for each proposed dependent variable (i.e., avoidance, coercion, disdain, and help-giving). Among the three stereotypical perceptions, only selfishness had a statistically significant direct effect on disdain (*ß* = 0.11, t = 2.05, *p* = 0.041). Table 3 presents the results for each dependent variable.

**Table 3:** Multiple mediation analysis results

	Mediators (M)											
	Anger			Fear			Shame			Disgust		
	ß	SE	t	ß	SE	t	ß	SE	t	ß	SE	t
Independent Variables (X)												
Incompetence (X1)	.10	.05	1.89 +	.08	.05	1.47	.47	.03	13.83***	.15	.03	3.98***
Selfishness (X2)	.22	.05	3.94***	-.04	.05	-.59	-.01	.03	-.17	.18	.03	2.16*
Immorality (X3)	.21	.06	3.73***	.23	.06	3.69***	.42	.04	11.30***	.75	.04	18.98***
Control Variables												
Gender (0 = Male, 1 = Female)	.02	.08	.59	.02	.08	.50	-.03	.05	-1.00	.02	.05	.59
Age	.06	.00	1.44	.07	.00	1.49	.07	.00	2.57**	.01	.00	.16
Religious Beliefs	-.03	.03	-.81	-.04	.03	-.92	-.03	.02	-.1.11	.00	.02	.16
Depression Level	.04	.01	1.00	.12	.01	2.73	-.02	.01	-.58	.02	.01	.56
Contact Experience (0 = Yes, 1 = No)	-.01	.09	-.23	.05	.09	1.21	-.02	.06	-.85	-.05	06	-1.91
Model Summary	${R}_{Model}^{2}$=.23, F (8, 543) = 20.41***			${R}_{Model}^{2}$=.09, F (8, 543) = 6.66***			${R}_{Model}^{2}$=.67, F (8, 543) = 138.83***			${R}_{Model}^{2}$=.62, F (8, 543) = 109.91***		
	Dependent Variables (Y)											
	Avoidance			Disdain			Coercion			Help-giving		
	ß	SE	t	ß	SE	t	ß	SE	t	ß	SE	t
Independent Variables (X)												
Incompetence (X1)	-.04	.05	-.70	-.07	.05	-1.17	.03	.05	.41	.11	.06	1.76
Selfishness (X2)	-.01	.05	-.19	.11	.05	2.05*	.00	.04	.04	.11	.05	1.80
Immorality (X3)	-.02	.07	-.31	-.02	.07	-.31	.01	.09	.09	.07	.08	.91
Mediators (M)												
Anger (M1)	.18	.04	4.21***	.24	.04	5.35***	.27	.04	5.79***	-.03	.05	-.57
Fear (M2)	.32	.04	8.14***	.22	.04	5.55***	.22	.04	5.26***	-.10	.04	-2.22*
Shame (M3)	.08	.07	1.16	.01	.07	.09	-.07	.07	.86	-.10	.08	-1.22
Disgust (M4)	.26	.07	3.90***	.38	.07	5.54***	.18	.07	2.49*	-.29	.08	-3.78***
Control Variables												
Gender (0 = Male, 1 = Female)	-.07	.07	-1.97*	-.06	.07	-1.64	.02	.07	.48	.08	.08	1.99*
Age	-.13	.00	-3.32**	-.14	.00	-3.52***	-.05	.00	-1.22	.18	.00	4.14***
Religious Beliefs	.05	.02	1.41	.05	.02	1.39	-.00	.02	-.08	-.06	.08	-.85
Depression Level	.03	.01	.86	.03	.01	0.74	.05	.01	1.21	-.01	.01	-.18
Contact Experience (0 = Yes, 1 = No)	.06	.09	1.72	.04	.09	1.10	.05	.08	1.30	.05	.10	1.22
Model Summary	${R}_{Model}^{2}$=.34, F (12, 539) = 23.20***			${R}_{Model}^{2}$=.30, F (12, 539) = 19.18***			${R}_{Model}^{2}$=.23, F (12, 539) = 13.25***			${R}_{Model}^{2}$=.13, F (12, 539) = 6.47***		

*N* = 552

^*^p < .05

^**^p < .01

^***^p < .001

### Path 3. Indirect effects of three stereotypical perceptions on each behavioral intention (X → M → Y)

#### Avoidance

Indirect effects were observed between the three subfactors of stereotype and avoidance. First, incompetence significantly increased disgust responses, which in turn were associated with a higher avoidant intention toward suicidal individuals (indirect effect: *ß* = 0.04, 95% CIs [0.01,0.057]). Selfishness was a significant predictor of both anger and disgust, with avoidance being influenced by significant indirect paths from selfishness through anger (indirect effect: *ß* = 0.03, 95% CIs [0.01,0.07]) and disgust (indirect effect: *ß* = 0.02, 95% CIs [0.00,0.05]). Additionally, the indirect effect of immorality on avoidance through fear (indirect effect: *ß* = 0.07, 95% CIs [0.03,0.12]), anger (indirect effect: *ß* = 0.04, 95% CIs [0.01,0.08]), and disgust (indirect effect: *ß* = 0.20, 95% CIs [0.09,0.30]) was significant.

#### Disdain

Incompetence significantly and positively predicted disgust (*ß* = 0.15, t = 3.98, *p* < 0.001), which in turn was associated with higher disdain (*ß* = 0.38, t = 5.55, *p* < 0.001). Supporting this path, the indirect effect of incompetence on disdain via disgust was significant (indirect effect: *ß* = 0.06, 95% CIs [0.02,0.10]). The results from a parallel mediation analysis indicated that selfishness was indirectly related to disdain tendencies through its relationships with anger (indirect effect: *ß* = 0.05, 95% CIs [0.02,0.09]) and disgust (indirect effect: *ß* = 0.03, 95% CIs [0.00,0.07]). Next, immorality did not have a significant direct influence on disdain intention toward suicidal individuals; however, it had indirect effects on disdain through fear (indirect effect: *ß* = 0.05, 95% CIs [0.02,0.09]), anger (indirect effect: *ß* = 0.05, 95% CIs [0.02,0.09]), and disgust (indirect effect: *ß* = 0.29, 95% CIs [0.16,0.41]).

#### Coercion

Coercion was not directly influenced by the three stereotype factors. However, it was directly affected by emotional responses such as fear (*ß* = 0.22, t = 5.26, *p* < 0.001), anger (*ß* = 0.27, t = 5.79, *p* < 0.001), and disgust (*ß* = 0.18, t = 2.49, *p* = 0.013). In contrast, no significant relationship was observed between shame and disdain (*ß* = -0.07, t = -0.86, *p* = 0.393). Incompetence significantly predicted disgust, which, in turn, was associated with higher coercion (indirect effect: *ß* = 0.03, 95% CIs [0.01,0.06]). Selfishness had significant indirect effects on coercion through anger (indirect effect: *ß* = 0.05, 95% CIs [0.02,0.08]) and disgust (indirect effect: *ß* = 0.01, 95% CIs [0.00,0.03]). Immorality positively predicted coercive intention toward suicidal persons only indirectly via fear (indirect effect: *ß* = 0.05, 95% CIs [0.02,0.09]), anger (indirect effect: *ß* = 0.06, 95% CIs [0.02,0.10]), and disgust (indirect effect: *ß* = 0.14, 95% CIs [0.03,0.24]).

#### Help giving

Stereotypes toward suicidal individuals—incompetence, selfishness, and immorality—had no direct effects on help-giving intention. Among the four mediators, less fear (*ß* = -0.10, t = -2.22, *p* = 0.027) and disgust (*ß* = -0.30, t = -3.78, *p* < 0.001) led to an increased likelihood of help-giving. However, anger (*ß* = -0.03, t = -0.57, *p* = 0.571) and shame (*ß* = -0.10, t = -1.22, *p* = 0.224) were not related to help-giving. The results also revealed significant indirect paths from the three negative stereotype factors to help-giving intentions. Incompetence (indirect effect: *ß* = -0.04, 95% CIs [-0.08, -0.02]) and selfishness (indirect effect: *ß* = -0.02, 95% CIs [-0.05, -0.00]) had negative and significant indirect effects on help-giving via disgust alone. Immorality was a significant predictor of fear and disgust, and higher reported fear (indirect effect: *ß* = -0.02, 95% CIs [-0.06, -0.00]) and disgust (indirect effect: *ß* = -0.21, 95% CIs [-0.33, -0.10]) were subsequently related to decreased help-giving intention.

## Discussion

This study demonstrated that the emotional responses elicited toward suicide attempters depend on the evaluative judgments made about them, revealing significant connections between cognition and emotion. Consistent with previous research on attribution theory and the controllability of the cause [22, 24], our findings reveal that perceiving suicide attempters as incompetent and irresponsible evokes feelings of shame and disgust. While prior studies primarily focused on anger or fear toward stigmatized individuals [17, 25], our research expands on these emotional reactions by incorporating shame and disgust as additional negative emotions directed toward stigmatized individuals.

In our study, incorporating shame and disgust as additional negative emotions deepens our understanding of stigma towards suicide attempters. These emotions are linked to perceptions of uncontrollability in mental health issues, aligning with attribution theory. They are intricately connected to social distancing and discriminatory behaviors against suicide attempters. Particularly, shame and disgust are powerful emotional responses in many cultures to behaviors considered taboo or deviant, like suicide, enhancing our study's cultural relevance [20]. By integrating these emotions into our model, we acknowledge the complexity and diversity of emotional reactions towards suicide attempters, significantly broadening the scope of our understanding in this area.

Moreover, this study examined common cognitive factors found in the suicide stigma scale, specifically the perceptions of selfishness and immorality. We discovered that when suicide attempters were evaluated as immoral and cruel, emotional responses previously unassociated with the label of incompetence were activated. Perceiving suicide attempters as immoral and cruel could simultaneously trigger fear, anger, disgust, and shame, in addition to the previously identified emotions of shame and disgust linked to incompetence and irresponsibility. The inclusion of shame and disgust as emotional reactions in our findings significantly contributes to the literature, providing a more comprehensive understanding of the negative emotions experienced toward stigmatized individuals.

**Fig. 2:**
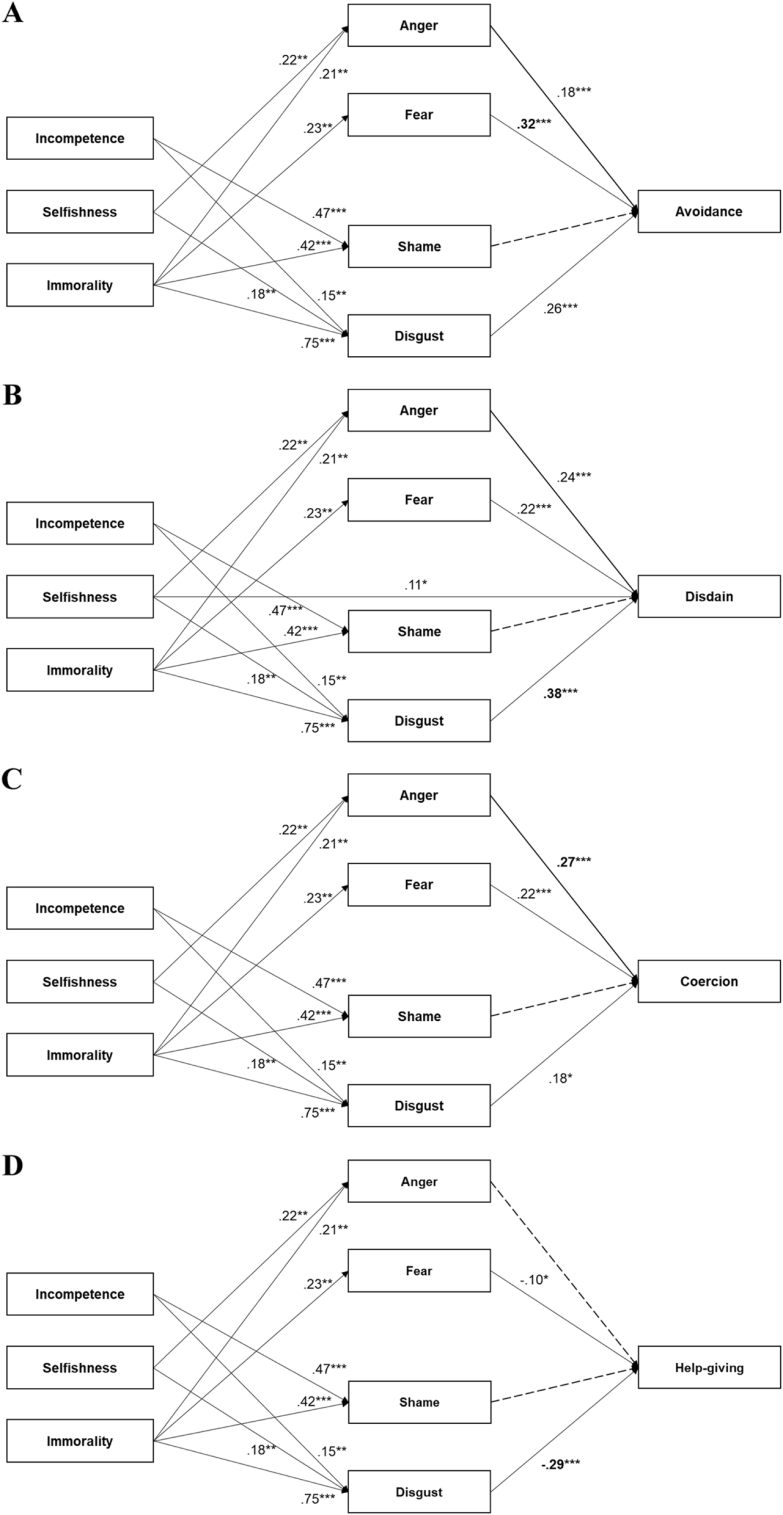
Multiple mediation displaying standardized coefficients for the direct and indirect paths from negative stereotypes through four types of emotional reactions to (**A**) avoidance, (**B**) disdain, (**C**) coercion, and (**D**) help-giving. *Note*. The dotted lines indicate the nonsignificant paths. The numbers in parentheses reflect the standardized coefficient in the absence of the mediating variables (i.e., the total effect). 95% CIs for indirect effects were computed using 5,000 bootstrap resamples and corrected for bias. Control variables: gender, age, religious beliefs, depression level, and contact experience with suicide *N* = 552, * *p* < .05. ** *p* < .01

A notable finding is that the behavioral response depends on the type of negative emotion elicited by stereotypes. Emotional responses arising from these perceptions predict intentions to either discriminate against or help suicidal individuals. Evaluating suicide attempters as incompetent elicits disgust, leading to avoidance, ignorance, and coercion (see Fig. 2). Perceiving selfishness evokes anger and disgust, resulting in discriminatory behaviors. When suicide attempters are considered immoral, fear, anger, and disgust are triggered, leading to avoidance, ignorance, and coercion. Help-giving intentions are influenced by perceiving immorality, which is mediated through fear and disgust. The more suicidal individuals are perceived as immoral and cruel, the higher the levels of fear and disgust, resulting in a decreased intention to provide help. These findings emphasize the importance of understanding and addressing these stereotypes to mitigate negative behavioral intentions and promote supportive actions toward suicidal persons. As previous studies suggest that stereotypes can inhibit prosocial actions [22, 24, 25], our research further dissects the emotional pathways—fear, anger, and disgust—that mediate this relationship, marking a distinctive contribution to the field. Addressing and reducing these stereotypes could help prevent discrimination and increase the likelihood of offering support to suicidal individuals.

Additionally, this study demonstrated that each emotional reaction has a notable impact on specific behavioral responses, as evidenced by effect size values. Avoidance was more sensitive to fear, disdain to disgust, and coercion to anger. These distinct emotional responses can guide the development of campaign strategies or educational materials for future suicide prevention initiatives. Furthermore, it is imperative to create suicide stigma reduction programs based on the findings of this study to effectively diminish the stigma surrounding suicide.

The cognitive-emotional-behavioral pathway model [16, 17] effectively predicts attitudes toward stigmatized individuals, particularly among those with limited direct contact experience with suicide attempters. Participants without direct contact experience are more susceptible to social stigma, which can influence their judgments of a specific target. In this context, cognitive modification can serve as an effective strategy for changing attitudes toward stigmatized individuals. Additionally, our study provides compelling evidence that enhancing suicide literacy education can help reduce suicide stigma, a critical factor in suicide prevention. Overall, this research underscores the importance of addressing stereotypes and emotional responses to promote more supportive attitudes toward suicidal individuals.

Interestingly, the shame experienced by individuals who have attempted suicide, due to their perceived incompetence and immorality, did not result in discriminatory behavior. According to previous studies [26–28], shame toward suicide attempters is frequently felt by their family members. The absence of a link between shame and discriminatory behavior may suggest that families may not feel compelled to treat the suicide attempter with indifference or social isolation. However, it is important to note that such shame can lead to the concealment of suicide and increase the likelihood of subsequent suicide risk [29]. Therefore, caution is necessary in addressing this issue.

Although this study did not specifically investigate the perception-emotion-behavioral intention pathway in close relationships involving suicide attempters, it underscores the crucial role of individuals surrounding the attempter in overcoming a suicidal crisis. Further research is required to examine and validate the intricate interplay between perception, emotion, and behavioral intentions in relationships with suicide attempters. Gaining a deeper understanding of the dynamics and influences on those close to the individual is essential for effective intervention and support in preventing suicide.

It is important to acknowledge that the intention to distance or discriminate against individuals who attempt suicide is not solely driven by cognitive information about suicide. Rather, it is shaped by the emotions evoked by such information. The findings of this study support previous meta-research by Rudolph et al. [17], which posited that pro- or antisocial behavior stems from emotional evaluations prompted by cognitive assessments. This study not only confirms a similar pathway but also highlights the additional roles of fear and disgust as emotions aroused by assessments of immorality. This study indicates that, in the context of social issues reflecting cultural normative evaluations, immoral factors play a more decisive role than other stigmatizing factors. This study sheds light on the continuous process of emotional reactions and behavioral intentions based on cognitive assessments of suicide attempters. Future research should explore the underlying attribution process or reasons for negative evaluations of suicide attempters, such as perceptions of immorality or selfishness. Investigating the causal process behind such stereotypical perceptions may contribute to the extension of attribution theory.

While research on the stigma of mental illness is more prevalent, studies focusing on the stigma of suicide remain limited. Despite the urgency of addressing suicide stigma, it is often understood within the context of mental illness stigma. This study reveals that, unlike mental illness stigma, suicide stigma involves strong evaluations of personal qualities, such as immorality, selfishness, and incompetence. Moreover, the emotional reactions triggered by suicide stigma differ, indicating a stronger influence of cultural norms compared to other stigmatized illnesses.

These findings emphasize the importance of considering cultural specificity in suicide stigma research. Although no studies have compared the cognitive and emotional aspects and subsequent behavioral reactions of suicide stigma across cultures, this study suggests that Eastern societies may exhibit distinct cognitive evaluations and emotional reactions compared to Western societies. Furthermore, within the same cultural context, negative stereotypes and emotional responses could vary depending on the target of suicide. Fu et al. [30] demonstrated a clear difference in perception between the suicides of famous individuals and ordinary individuals. Additionally, given that the degree of emotional activation can affect the level of proactive behavior response [31], it would be meaningful to examine how varying degrees of emotional activation, high-arousal emotions such as anger and hatred, and low-arousal emotions such as indifference, impact support for policies related to the protection and welfare of suicide attempters. To develop tailored suicide prevention strategies, detailed research exploring the antecedents and ripple effects of suicide stigma is essential and should continue.

Our study underscores the importance of reducing negative stereotypes toward suicide attempters, which may lead to a decrease in discriminatory behaviors and an increase in the likelihood of providing help. Mental health professionals and suicide prevention advocates should focus on increasing suicide literacy education to reduce the stigma surrounding suicide. Furthermore, cognitive modification could be an effective strategy for changing attitudes toward stigmatized individuals. By reducing negative stereotypes, we can foster a more compassionate and supportive society for those struggling with suicidal thoughts or who have attempted suicide.

### Limitations and implications

This study has some limitations. First, the cross-sectional design constrains the capacity to infer causal connections between variables. To enhance the explanatory power and establish causality, longitudinal or experimental designs would be needed. Another limitation is that this study did not thoroughly examine the influence of sympathy, an important aspect of suicide stigma in Korean society. As negative stereotypes did not predict sympathy in this study, we did not specifically discuss their effect on behavioral responses toward suicidal individuals. Previous research [17] has identified sympathy as a crucial variable in predicting supportive behavior. People who feel sympathy for suicidal individuals treat them with pity. Sympathy positively influences the provision of assistance and may moderate the impact of emotional reactions triggered by negative stereotypes. Future studies should consider sympathy as an ambivalent emotion in relation to suicidal individuals and explore its effects in greater detail.

The present findings offer significant contributions to the literature on stigma and suicide prevention. First, emotion is an important factor influencing behavioral intention. Our study found no direct effect between stereotypes and behavioral intention, which suggests that it is important to consider the role of emotion when discussing stigma reduction strategies. Previous studies on stigma reduction, based on attribution theory, have prioritized the cognitive aspect of stigmatized individuals over the impact of emotion [21, 32]. For example, factors such as onset controllability or responsibility have been used to determine discriminatory or supportive behaviors toward a stigmatized target. However, this study indicates that people’s reluctance to interact with suicidal individuals stems from the unpleasant emotions they associate with them and that these emotional reactions are more complex than previously thought.

The results of this study also have practical implications. First, interventions aimed at reducing negative stereotypes about suicidal individuals may help decrease negative emotional responses, thereby contributing to more supportive and helpful behaviors. Second, mental health professionals, educators, and policymakers should consider addressing emotional responses to suicide alongside stereotypes to promote more compassionate attitudes and behaviors toward suicidal individuals. Future research should explore other potential mediators and moderators in the relationship between stereotypes, emotional responses, and behavioral intentions. Additionally, examining the role of cultural factors and individual differences in shaping these relationships could provide further insights and help tailor interventions to specific populations.

## Conclusions

This research sheds light on the complex relationships between cognitive evaluations, emotional reactions, and behavioral intentions in relation to individuals who have attempted suicide, thereby contributing to the broader discourse on suicide stigma within society. Our study distinctly shows how negative stereotypes trigger specific emotional responses, subsequently shaping intentions and actions. This underscores the critical necessity of tackling both cognitive and emotional dimensions in our endeavors to dismantle stigma and encourage supportive behavior.

Our findings particularly underscore the vital role of cognitive modification and suicide literacy education in diminishing stigma and fostering a supportive and inclusive community. This work lays the groundwork for mental health professionals, educators, and policymakers to develop and implement interventions that are grounded in a deep understanding of the emotional and cognitive dimensions of suicide stigma. Moving forward, there is a clear call to action for society to uphold a commitment to empathy, understanding, and support for those grappling with suicidal thoughts. Targeted interventions and educational initiatives have the potential to reshape perceptions, alter emotional responses, and cultivate a society that stands united in support of individuals during their most vulnerable moments.

Through this study, we have illuminated a path forward, providing evidence-based insights that are crucial for guiding future efforts in suicide prevention and stigma reduction. The journey towards eradicating suicide stigma is a collective one, and with continued research and dedication, we can work towards a future where support and compassion prevail.

## Data Availability

The data that support the findings of this study are available on request from the corresponding author.
